# Cloning and Expression of the Neuropeptide F and Neuropeptide F Receptor Genes and Their Regulation of Food Intake in the Chinese White Pine Beetle *Dendroctonus armandi*

**DOI:** 10.3389/fphys.2021.662651

**Published:** 2021-06-18

**Authors:** Bin Liu, Danyang Fu, Haiming Gao, Hang Ning, Yaya Sun, Hui Chen, Ming Tang

**Affiliations:** ^1^College of Forestry, Northwest A&F University, Xianyang, China; ^2^State Key Laboratory for Conservation and Utilization of Subtropical Agro-Bioresources, Guangdong Key Laboratory for Innovative Development and Utilization of Forest Plant Germplasm, College of Forestry and Landscape Architecture, South China Agricultural University, Guangzhou, China

**Keywords:** *Dendroctonus armandi*, Neuropeptide F, Neuropeptide F receptor, food intake, energy metabolism, RNA interference

## Abstract

Neuropeptide F (NPF) is an important signaling molecule that acts as a neuromodulator to regulate a diversity of physiological and behavioral processes from vertebrates to invertebrates by interaction with NPF receptors, which are G protein-coupled receptors (GPCR). However, nothing is known about NPF in Chinese white pine beetle, *Dendroctonus armandi*, a destructive pest of natural and coniferous forests in the middle Qinling Mountains of China. We have cloned and characterized cDNAs encoding one NPF precursor and two NPF receptors in *D. armandi* and made bioinformatics predictions according to the deduced amino acid sequences. They were highly similar to that of *Dendroctonus ponderosa.* The transcription levels of these genes were different between larvae and adults of sexes, and there were significant differences among the different developmental stages and tissues and between beetles under starvation and following re-feeding states. Additionally, downregulation of NPF and NPFR by injecting dsRNA into beetles reduced their food intake, caused increases of mortality and decreases of body weight, and also resulted in a decrease of glycogen and free fatty acid and an increase of trehalose. These results indicate that the NPF signaling pathway plays a significant positive role in the regulation of food intake and provides a potential target for the sustainable management of this pest.

## Introduction

Neuropeptides play significant regulatory roles in both vertebrates and invertebrates. Neuropeptide Y (NPY) is not only one of the most widespread neuropeptides in the central nervous system (CNS) of vertebrates (Allen, 1990) but also an important molecule to regulate diverse physiological and behavioral processes (Cerdá-Reverter and Larhammar, 2000). Neuropeptide F (NPF) is a homolog of NPY found in invertebrates, which is highly similar to NPY in structure and function but differs from NPY in having a phenylalanine (F) instead of a tyrosine (Y) at the C-terminus (Maule et al., 1991; de Jong-Brink et al., 2001; Roller et al., 2008; Cui and Zhao, 2020; Yeoh et al., 2017). NPFs have been identified in 30 Coleopteran species (Pandit et al., 2019), which are evolutionarily well conserved. The NPFs of most insects contain more than 28 residues (generally 28–45 amino acids) and are characterized by a RxRFamide consensus sequence at the carboxy terminus. However, some short peptides of 8–10 amino acids in length were found to be encoded by another gene, the short NPF (sNPF), characterized by an M/T/L/FRF amide carboxyterminal motif (Nässel and Wegener, 2011). NPFs operate through interacting with the NPF receptors, which belong to members of the G protein-coupled receptor (GPCR) superfamily. The NPF/NPFR signaling system provides a new paradigm for exploring the central regulation of cooperative and behavioral processes (Wu et al., 2003). NPFRs have been identified in *Drosophila* and *Anopheles* (Garczynski et al., 2002, 2005) and are also predicted in various other insect species (Caers et al., 2012). NPFs have a significant role in regulating feeding and foraging behaviors (Shen and Cai, 2001; Wu et al., 2003; Fadda et al., 2019). As a matter of fact, regulation of feeding was the first observed role of NPF, and most functional insights have been mainly obtained in studies with *Drosophila melanogaster* (Brown et al., 1999; Wu et al., 2005a, b; Wang et al., 2013). For example, the NPF signaling system has an effect on consuming noxious food in larvae of *D. melanogaster* (Wu et al., 2005b). Overexpression of NPFR in fly larvae causes an augmentation of noxious food uptake, while RNAi-mediated knockdown of NPFR shows the opposite phenotype (Wu et al., 2005b). Similarly, NPF signaling also stimulates feeding behavior under cold conditions in Drosophila larvae (Lingo et al., 2007).

Additionally, NPFs are also involved in many other functions, such as sexual and male characteristic courtship behavior (Lee et al., 2006; Kim et al., 2013), ethanol sensitivity (Wen et al., 2005), learning and memory (Krashes et al., 2009), aggression (Dierick and Greenspan, 2007), locomotor activity, and circadian rhythm (Hermann et al., 2012; Erion et al., 2016). However, the role of NPF in other insects has been rarely explored and has mainly focused on the functions related to feeding behavior. The association between NPF signaling and feeding-related processes, as documented in *Drosophila*, has also been observed in *Locusta migratoria*, with starvation causing an increase of *LmiNPF1* expression level, and downregulation of *LmiNPF1* notably reduced food intake (Tan et al., 2019). Furthermore, the NPF expression levels in *Acyrthosiphon pisum* were significantly higher in starved aphids than in satiated aphids, and RNAi-mediated knockdown of NPF in adult aphids markedly inhibited feeding behavior (Li et al., 2018). Moreover, NPF transcript levels of *Schistocerca gregaria* were significantly upregulated in starved animals compared with feeding animals. Injection of trNPF in locust adults caused an increase in food intake, while RNAi knockdown showed the opposite effect (Van Wielendaele et al., 2013). The NPF signaling system has also been shown to regulate feeding and growth development in *Bombyx mori*, where dsRNA-mediated knock-down of BomNPFR leads to a significant reduction in food intake and body weight (Deng et al., 2014).

*Dendroctonus armandi* Tsai and Li (Coleoptera: Curculionidae: Scolytinae) is a destructive pest of natural and coniferous forests in the middle Qinling Mountains of China, which only attacks healthy *Pinus armandi*, devastating the forest ecological system and incurring heavy economic losses (Chen and Tang, 2007; Hu et al., 2013). Although the NPFs have been explored in some insect species, there are no reports on the functional roles of NPF in bark beetles. In this study, to investigate whether NPF signaling in *D. armandi* is involved in food intake or not, we identified and cloned cDNAs encoding a NPF precursor and two NPFRs from *D. armandi*, which were used for further investigations of related functions. These information will serve as a considerable step forward to provide a potential molecular target for the sustainable management of bark beetles.

## Materials and Methods

### Insect Sample Preparation

*D. armandi* was collected from infested *Pinus armandi* trees at the Huoditang Experimental Forest Station, which is located on the southern slope of the middle Qinling Mountains, in Shaanxi, China (33°18′N, 108°21′E). The newly infected *P. armandi* was transported to the laboratory from the sampling point after felling, where emerged adults and larvae were collected. They were reared in glass dishes (90 × 15 mm) at 20 ± 1°C, 70% relative humidity, and in the dark in an artificial climate cabinet of the laboratory using the meridic diet as described previously. Adults were sexed by the shape of external genitals and other male-characteristic auditory cues (Dai et al., 2014; Zhao et al., 2017).

### Total RNA Extraction and cDNA Synthesis

Total RNA was isolated from three development stages (larvae, pupae, and adults) using the UNlQ-10 Column Trizol Total RNA Isolation Kit (Sangon Biotech, Shanghai, China). Total mixed RNA for cDNA was synthesized using the Fast King RT reagent Kit (with gDNase) (Tiangen, China) and then stored at −20°C until use.

### Identification of cDNAs Encoding NPF and NPFRs in *D. armandi*

#### cDNA Amplification and Cloning

The synthesized cDNA obtained from the samples was used as a template for PCR. Each pair of specific primers (Supplementary Table 1) was designed in Primer Premier 5.0, based on *NPF* and *NPFR* sequences of other related insect species from NCBI^1^ GenBank. All the PCR amplifications were performed with a C1000 thermocycler (Bio-Rad Laboratories, Hercules, CA, United States) in a final reaction mixture of 20 μl, containing 1 μl cDNA (1:4 dilution), 0.25 μl of each primer, and 2 × EcoTaq PCR SuperMix (TransGen, Beijing, China). The sequenced PCR products were manually edited using the DNAMAN software.

#### 5′, 3′ RACE and Cloning of Full-Length cDNA

cDNA-specific primers for 5′ and 3′ RACE (Supplementary Table 1) were designed according to the obtained sequence. Touchdown PCR (annealing temperatures: 65–55°C) was used to improve the amplification specificity of the 5′-UTR and 3′-UTR sequences. The amplified products were cloned and sequenced as followed by previous description “cDNA amplification and cloning.” To obtain the full-length sequences, we designed specific primers containing the putative initiation and terminator codons (Supplementary Table 1).

#### Analysis of cDNA Sequences

The three cDNA sequences obtained were submitted in the GenBank, and accession numbers are listed in Table 1. The open reading frames (ORFs) of full-length cDNA sequences were obtained using ORF Finder^2^. Multiple sequence alignment of sequences was carried out in *DNAMAN6.0*. Molecular mass (kDa) and isoelectric points were determined in the ProtParam tool (Gasteiger et al., 2005). The putative signal peptide was predicted using Signal P 4.1 Server^3^. All putative *D. armandi* NPF and NPFR proteins were predicted for subcellular localization using the TARGETP tool^4^ (Emanuelsson et al., 2000) with the default parameters. TMHMM v. 2.0^5^ was used to visually display predictions of topological and transmembrane domains. The phylogenetic trees were constructed by software MEGA 6.0 (Tamura et al., 2013) using the maximum likelihood method with a Whelan and Goldman (WAG) model and a gamma parameter value. The support for each node of bootstrap was estimated by a bootstrap program after 500 replicates.

**TABLE 1:** Physicochemical properties and cellular localization of *D. armandi* NPF and NPFR proteins.

Gene name	Accession no.	ORF size (aa/bp)^a^	MW (KDa)^a^	IP^a^	Signal peptide prediction^b^
NPF	MT939856	133/402	15.55	9.97	SP 0.957 mTP 0.051 other 0.048
NPFR1	MT939857	418/1,257	47.88	8.63	SP 0.383 mTP 0.061 other 0.769
NPFR2	MT939858	400/1,203	45.85	8.44	SP 0.383 mTP 0.061 other 0.769

*ORF, open reading frame; MW, molecular weight; IP, isoelectric point; mTP, mitochondrial targeting peptide; SP, secretory pathway signal peptide. ^a^As predicted by the PROTPARAM program. ^b^As predicted by TARGET P1.1.*

### Expression Patterns of NPF and NPFR Genes in Different Life Stages, Tissues, and Treatments

#### Insects Sampling and Treatments for RT-qPCR

*D. armandi* larvae were separated into two substages: larvae (eating on host phloem for development) and mature larvae (cease feeding). Pupae were separated into two substages: early pupae (newly transformed from larvae) and late pupae (approach to become adults). We separated the adults into three substages: teneral adults (light body color), emerged adults, and feeding adults (invading the bark) (Dai et al., 2014). The heads, thoraxes, foreguts, midguts, hindguts, pheromone glands, and body fat of emerged adults, and heads, thoraxes, foreguts, midguts, hindguts, and body fat of larvae were dissected for measurement of expression level in tissues. Samples were collected in triplicate, killed by submerging beetles in liquid nitrogen, and then stored at −80°C until use.

The males and females of emerged adults were divided into eight groups, and larvae were divided into seven groups. One group of collected insects were killed at 0 h of feeding, which were used as the control. Each of the other groups of emerged adults was immediately placed in glass dishes (90 × 15 mm) with normal food for 24 and 48 h in the artificial climate cabinet. After 48 h of normal feeding, the adults and larvae were reared without food and starved for 72 and 48 h, respectively. Then, the alive insects were subsequently re-fed for 24 h after starvation treatment. The experiments of each treatment for real-time PCR analyses were explored in three biological replicates independently, with 15 adults of each sex and 20 larvae at each repeat.

#### RT-qPCR

The frozen samples were used for RNA extraction and cDNA synthesis following the above description “Total RNA Extraction and cDNA Synthesis.” All samples were placed in the CFX-96^TM^ real-time PCR Detection System (Bio-Rad, CA, United States) for RT-qPCR. The β-actin (GenBank accession no. KJ507199.1) and CYP4G55 (GenBank accession no. JQ855658.1) sequence of *D*. *armandi* was used as the internal control (Vandesompele et al., 2002; Dai et al., 2015). RT-qPCR primers were designed using Primer Premier 5.0 according to the obtained sequences (Supplementary Table 1). The amplification efficiency of each transcript was analyzed with relative standard curves at a different dilution series (1.0, 10^–1^, 10^–2^, 10^–3^, and 10^–4^) of cDNAs, and the efficiency values of the primers were analyzed by 100 ± 5%. The reaction mixture (20 μl) contained 1 μl of each primer, 2.5 μl of cDNA (diluted four times), 5.5 μl of ddH_2_O, and 10 μl of 2 × SYBR Premix Ex Taq (Roche Diagnostics GmbH, Mannheim, Germany). The specificity of qPCR primers was estimated by the melting curve analysis. Three technical replicates of each treatment contained three biological replicates, which were performed to verify reproducibility. The relative expression level of each gene was analyzed by the 2^–ΔΔCt^ method (Livak and Schmittgen, 2001, 2008).

### RNA Interference

#### The dsRNA Synthesis and Injection

The synthesis of dsRNA was prepared using the T7 Ribo-MAXTM Express RNAi System (Promega, Madison, MI, United States). The primers (Supplementary Table 1) used for RNAi were designed according to the obtained sequences. The final dsRNA products were diluted to 1,000 ng/μl with DEPC water. Before injection, emerged adults and larvae were anesthetized by placing on a tray in an ice bath for 20 min. Afterward, each of emerged adults was injected with 0.2 μl dsRNA solution and larvae were injected with 0.l μl visa Hamilton Microliter^TM^ syringes (700 series, RN) with 32 G sharp-point needles (Hamilton, Bonaduz, Switzerland). Non-injected insects and insects injected with DEPC-treated water were used as controls in all experiments. Then, beetles were kept in an artificial climate cabinet under the feeding or starvation condition. Each treatment group contained 40 individuals, and 6 individuals of each treatment and after 72 h were collected and then stored at −80°C until qRT-PCR. Each treatment group contained three biological replicates.

#### Survival Test and Body Weight Measurement

After 72 h of dsRNA injection, the beetles were kept at room temperature for 1 h, and animals that did not move were considered to be dead (Fu et al., 2019). Adults and larvae mortality were quantified under the different treatments and control conditions to determine their effect at different time points (12, 24, 36, 48, 60, and 72 h). The body weight of each alive sample was immediately measured by an electronic balance (d = 0.0001 g, Tianjin, AL204; Mettler-Toledo Ltd., China). The measurement included three replicates.

#### Determination of Glycogen, Free Fatty Acid, and Trehalose

For emerged adults and larvae at 72 h after injection, we measured three physiological indices in each treatment group, including the content of glycogen, trehalose, and free fatty acid, *via* relevant biochemical methods. Three biological replicates (six beetles for one replicate) were performed for each measurement. Whole-body homogenates of each group were used to measure glycogen, trehalose, and free fatty acid, respectively. The three content levels were measured with a spectrophotometer (UV-1800PC, Shanghai Mapada Instrument Co., Ltd., Shanghai, China) using the relevant kit (TY-2-Y for glycogen, FFA-1-W for free fatty acid, and HT-2-Y for trehalose, respectively; SuzhouComin Biotechnology Co. Ltd., Jiangsu, China).

### Statistical Analysis

All data were statistically analyzed with SPSS Statistics 19.0 (IBM, Chicago, IL, United States). Significant differences between different treatments were determined using *post hoc* Tukey tests through one-way ANOVA. Student’s *t*-test was employed to perform the two-sample analyses. Graphs were plotted with Prism 6.0 (GraphPad Software, CA, United States).

## Results

### Sequencing and Bioinformatic Analysis

We successfully sequenced cDNAs encoding NPF and NRFRs from *D. armandi* with full-length sequences, and *NPF* cDNA encoded 133 amino acids with a predicted molecular mass of 15.55 kDa and isoelectric point of 9.97 (Table 1). Two splicing variants encoded by the *NPFR* gene were identified, with NPFR1 and NPFR2 comprising 418 and 400 amino acids, respectively. The cellular localization of these proteins shows that NPF is a secretory protein, whereas NPFR1 and NPFR2 are membrane proteins (Table 1).

The NPF precursor of *D. armandi* consisted of 133 amino acids, and the first 25 amino acids were predicted to be an N-terminal signal peptide, followed by the mature NPF (Supplementary Figure 1). The NPF prepropeptide contained processing sites at the C-terminal F residue (RPRFGKR), including an amidation site (G) and followed by a dibasic cleavage site (KR) (Supplementary Figure 1). The protein sequence of NPFR was predicted to have seven transmembrane domains and showed the typical characteristic of the rhodopsin-like GPCR family (Supplementary Figure 2). Further identities showed that DaNPF and DaNPFRs had the highest degree similarity with that of fellow Coleoptera member *Dendroctonus ponderosa* (Table 2). The phylogenetic tree of NPF (Supplementary Figure 3) and NPFRs (Supplementary Figure 4) revealed that these proteins were clustered with the Coleoptera group. Although only a few NPF sequences were listed in this study, we could also find more relevant information of protein sequences with other Coleoptera species (Veenstra, 2019).

**TABLE 2:** Identity of NPF and NPFR genes from *D. armandi* with relevant gene sequences in other insect species.

Gene	BLAST matches in GenBank			
Name	Species	Name	Accession number	Identity^a^
NPF	*Dendroctonus ponderosae*	Uncharacterized protein	XP_019762446.1	91
	*Rhynchophorus ferrugineus*	Neuropeptide F	QGA72566.1	66
	*Sitophilus oryzae*	Uncharacterized protein	XP_030767369.1	59
NPFR1	*Dendroctonus ponderosae*	Neuropeptide F receptor isoform X1	XP_019756679.1	96
	*Sitophilus oryzae*	Neuropeptide F receptor	XP_030754183.1	74
	*Rhynchophorus ferrugineus*	Neuropeptide f receptor 1	QGA72501.1	73
NPFR2	*Dendroctonus ponderosae*	Neuropeptide F receptor isoform X2	XP_019756681.1	97
	*Anoplophora glabripennis*	Neuropeptide F receptor	XP_018564523.1	79
	*Sitophilus oryzae*	Neuropeptide F receptor	XP_030754183.1	76

*^a^ As predicted by BLAST (http://www.ncbi.nlm.nih.gov).*

### RT-qPCR

#### Analysis of NPF, NPFR1, and NPFR2 Expression in Different Developmental Stages and Tissues

**FIGURE 1:**
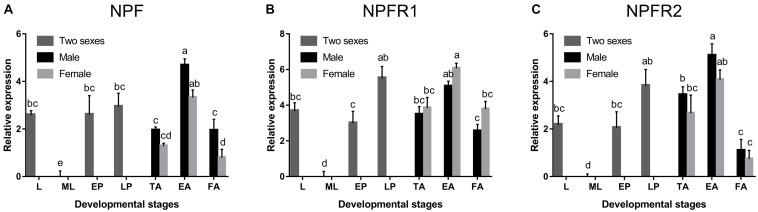
Relative mRNA expression levels of NPF **(A)**, NPFR1 **(B)**, and NPFR2 **(C)** in different developmental stages of *D. armandi*. The relative expression levels were normalized with β*-actin* and *CYP4G55* using the expression levels in the mature larvae for calibration. Different lowercase letters indicate significant differences at the 0.05 level. All values are mean ± *SE*, *n* = 3. L, larvae; ML, mature larvae; EP, early stage pupae; LP, late stage puage; TA, teneral adults; EA, emerged adults; FA, feeding adults.

NPF, NPFR1, and NPFR2 were broadly expressed in all developmental stages of *D. armandi*, but with different patterns. All of them were highly expressed in emerged adults, followed by pupae, and the lowest in mature larvae (Figure 1). Compared with NPFR1 and NPFR2, the expression levels of NPF in the larval stage and two pupal stages were not significantly different (Figure 1A). In the adult stage, the expression levels of NPF and NPFR2 in males were higher than in females (Figures 1A,C), while NPFR1 showed the opposite result (Figure 1B).

**FIGURE 2:**
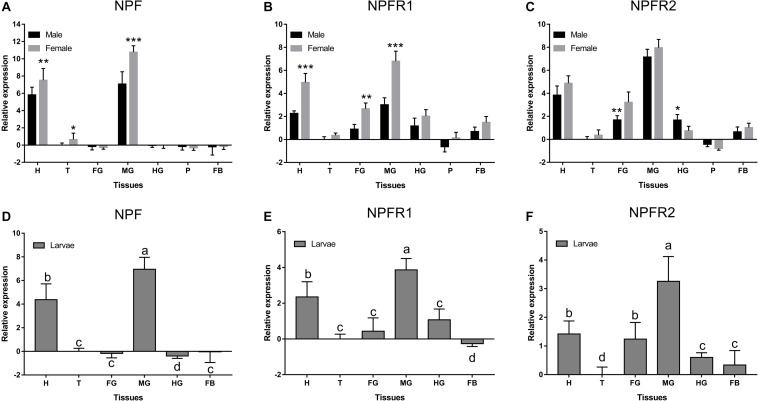
Relative expression levels of emerged adults of NPF **(A)**, NPFR1 **(B)**, NPFR2 **(C)**, and larvae of NPF **(D)**, NPFR1 **(E)**, and NPFR2 **(F)** in different tissues of *D. armandi.* The relative expression levels were normalized with β*-actin* and *CYP4G55* using the expression levels in the thorax for calibration. Different lowercase letters indicate significant differences at the 0.05 level. The asterisk indicates a significant difference between female and male expression levels (*P < 0.05, **P < 0.01, ***P < 0.001, independent Student’s *t*-test). All values are mean ± *SE*, *n* = 3. H, head; T, thorax; FG, foregut; MG, midgut; HG, hindgut; P, pheromone gland; FB, fat body.

NPF, NPFR1, and NPFR2 were expressed at different levels and with occasional sex differences among the different tissues (Figure 2). NPF was highly expressed only in the head and midgut of adults (Figure 2A) and larvae (Figure 2D), while NPFR1 (Figures 2B,E) and NPFR2 (Figures 2C,F) were expressed in different tissues. All of them were highly expressed in midgut, followed by head in adults and larvae. NPF was more highly expressed in females than in males in head, thorax, and midgut (Figures 2A,D). NPFR1 and NPFR2 were highly expressed in head, foregut, and midgut, with a strikingly higher expression in females than in males among the three tissues. Specifically, the expression of NPFR2 in the hindgut of males was significantly higher than that of females (Figure 2C).

#### Analysis of NPF, NPFR1, and NPFR2 Expression in Starvation and Re-feeding Assays

**FIGURE 3:**
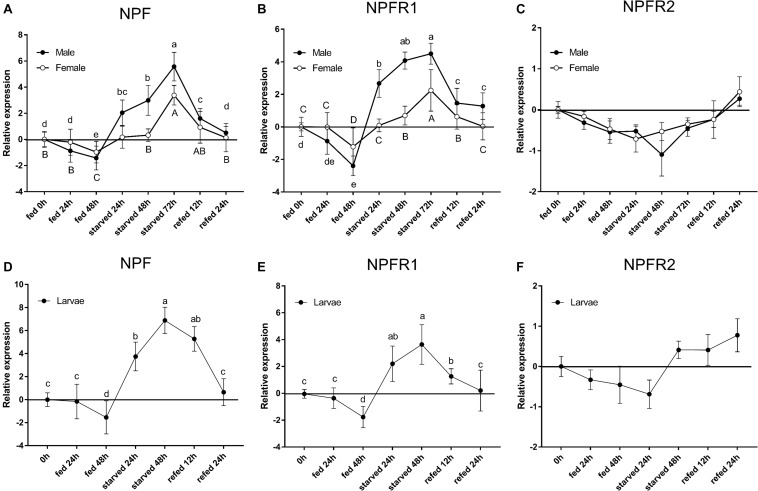
Relative expression levels of emerged adults of NPF **(A)**, NPFR1 **(B)**, NPFR2 **(C)**, and mature larvae of NPF **(D)**, NPFR1 **(E)**, and NPFR2 **(F)** in *D. armandi* after starvation and subsequent re-feeding treatment. The relative expression levels were normalized with β*-actin* and *CYP4G55* using the expression levels in 0 h for calibration. Different letters indicate significant differences at the 0.05 level (uppercase for males, lowercase for females and uppercase for males and larvae, no letter means no significant difference among all time points). All values are mean ± *SE*, *n* = 3.

Expression of three genes analyzed in adults and larvae showed a different response to starvation stress (Figure 3). Compared with the feeding groups, the NPF and NPFR1 expression levels in adults (Figures 3A,B) and larvae (Figures 3D,E) were highly upregulated in the starvation groups and reached the highest level at 72 h. Moreover, during the refeeding experiment after food deprivation, the NPF and NPFR1 expression levels showed a steady decline and then returned to the original level after refeeding for 24 h. The expression levels of NPF (Figure 3A) and NPFR1 (Figure 3B) in male adults were lower than those in female adults at 48 h after feeding, and the highest at 72 h after starvation. However, the expression level of NPFR2 was not affected in either the starvation or the following refeeding assay (Figures 3C,F).

### Efficiency Analysis of RNAi on DaNPF and DaNPFRs

#### Effect of dsRNA Injection on NPF and NPFR Expression Level

**FIGURE 4:**
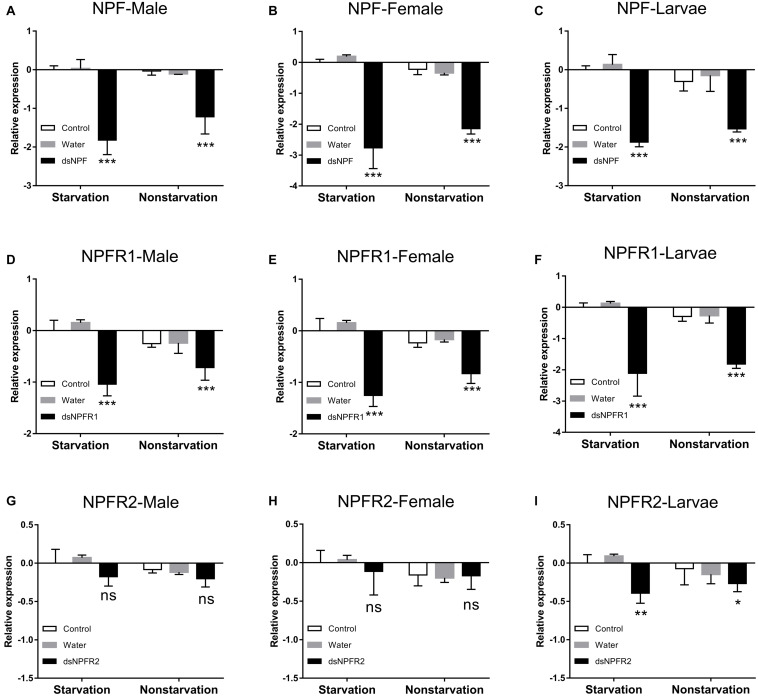
Knockdown of NPF-Male **(A)**, NPF-Female **(B)**, NPF-Larvae **(C)**, NPFR1-Male **(D)**, NPFR1-Female **(E)**, NPFR1-Larvae **(F)**, NPFR2-Male **(G)**, NPFR2-Female **(H)**, and NPFR2-Larvae **(I)** expression levels in *D. armandi* after injecting dsRNA for 72 h in the state of starvation and non-starvation. The asterisk indicates a significant difference between control and RNAi treatments according to Student’s *t*-test (*P < 0.05, **P < 0.01, ***P < 0.001, ns, not significant). All values are mean ± *SE*, *n* = 3.

In both the starvation and the feeding groups, compared with the other two control groups, the expression level of NPF (Figures 4A–C) and NPFR1 (Figures 4D–F) in adults and larvae was significantly downregulated at 72 h after dsRNA injection, respectively, and the expression level of NPF and NPFR1 in the starvation group was decreased more than the feeding group; after 72 h of dsNPFR2 injection, there was no obvious change in the male (Figure 4G) and female (Figure 4H) adults, but the expression level of the larvae was downregulated in both the starvation group and the feeding group (Figure 4I).

#### Effect of dsRNA Injection on Mortality and Body Weight

**FIGURE 5:**
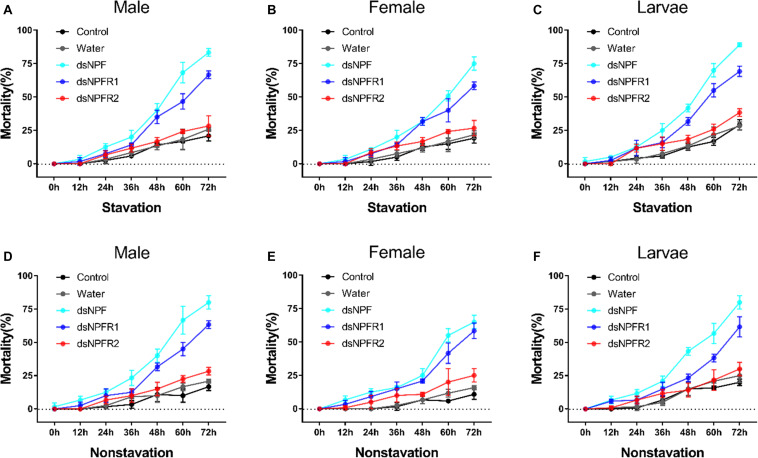
Mortality responses of RNAi *D. armandi* male **(A)**, female **(B)**, and larvae **(C)** in starvation and male **(D)**, female **(E)**, and larvae **(F)** in non-starvation to different time points. Mortality responses of dsRNA-treated, water-injected, and non-injected in *D. armandi* to different time points (0, 12, 24, 36, 48, 60, and 72 h). All experiments were analyzed by Student’s *t*-test, and in **(A–F)**, experimental beetles significantly differed (*P* < 0.01) from two controls.

In both the feeding and starvation groups, the mortality of the dsRNA injection in adults and larvae was higher than that of the non-injected and water-injected controls (Figure 5). The mortality significantly increased when the adults and larvae were injected with dsNPF and dsNPFR1 from 0 to 72 h. However, in the dsNPFR2-injected group, there was no significant change. The highest mortality was found after injection of dsNPF at 72 h. Moreover, the mortality of larvae was the highest and female adults was the lowest, 89.2 and 58.3%, respectively (Figures 5C,E). However, the mortality of the starvation group was significantly higher than that of the feeding group (Figure 5).

**FIGURE 6:**
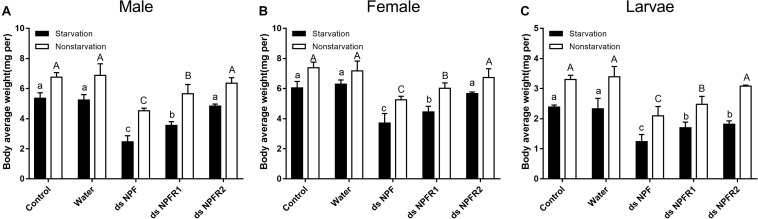
Effect of dsNPF, dsNPFR1, and dsNPFR2 on body average weight of *D. armandi*. Samples were collected and assayed at 72 h after injection. In the RNAi experiments, body average weight of male **(A)**, female **(B)**, and larvae **(C)** in the state of starvation and non-starvation was analyzed. Different letters indicate significant differences at the 0.05 level (uppercase for non-starvation, lowercase for starvation). All values are mean ± *SE*, *n* = 3.

Compared with the two control groups, the average weight of adults and larvae significantly decreased after 72 h of injection of dsNPF and dsNPFR1 in the starvation and feeding groups; among them, the male and the female adults had the most and the least weight loss, with 54.2 and 28.9%, respectively (Figures 6A,B). In particular, the average body weight of animals injected with dsNPFR2 did not change significantly, except for the larvae in the starvation group. Compared with the feeding group, the average weight of adults and larvae in the starvation group showed more reductions after dsRNA injection (Figure 6). These results indicate that the RNAi mediated downregulation of NPF inhibited food intake of beetles.

#### Effects of dsRNA Injection on Energy Metabolism

**FIGURE 7:**
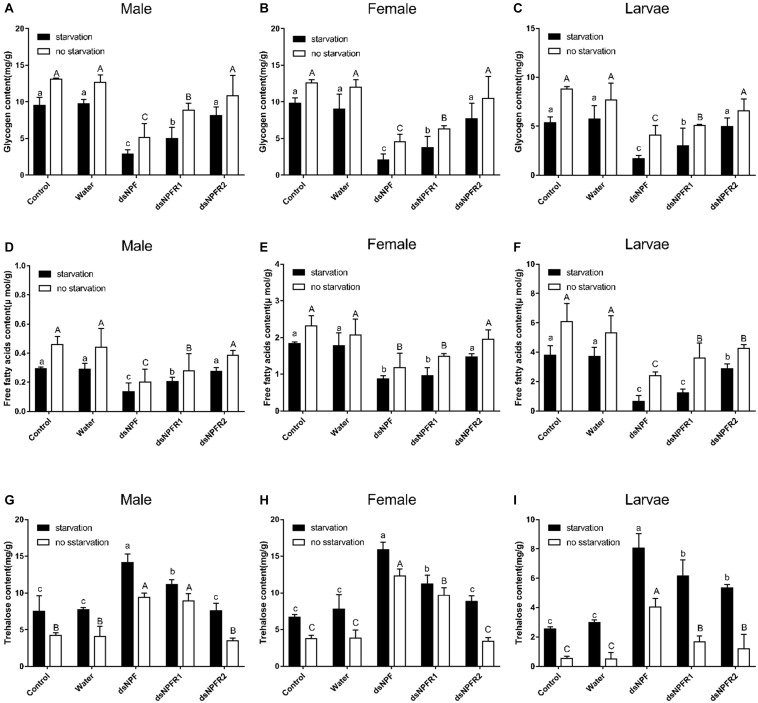
Effect of dsNPF, dsNPFR1, and dsNPFR2 on energy metabolism of *D. armandi*. Samples were collected and assayed at 72 h after injection. Whole-body homogenates were used to measure glycogen of male **(A)**, female **(B)**, and larvae **(C)**, Free fatty acids of male **(D)**, female **(E)**, and larvae **(F)** and trehalose contents of male **(G)**, female **(H)**, and larvae **(I)** in the state of starvation and non-starvation. Different letters indicate significant differences at the 0.05 level (uppercase for non-starvation, lowercase for starvation). All values are mean ± *SE*, *n* = 3.

Compared with the two control groups, the glycogen and free fatty acid contents of adults and larvae were significantly decreased after injection of dsNPF and dsNPFR1 for 72 h in the starvation group and the feeding group, and the largest decline was in the dsNPF injection group (Figure 7). In particular, there was no significant change in the glycogen content of the larvae injected with dsNPFR2, but the free fatty acid content of the larvae injected with dsNPFR2 was decreased (Figure 7F). More interestingly, compared with the two control groups, the trehalose content of adults and larvae increased significantly at 72 h after injection of dsNPF and dsNPFR1, respectively, but there was no obvious change in the trehalose content of male and female adults injected with dsNPFR2 (Figure 7). Compared with the feeding group, the content of glycogen and free fatty acid decreased in the starvation group after injection of dsRNA. On the contrary, the content of trehalose increased (Figure 7).

## Discussion

Most NPFs are encoded by one gene copy per species, based on genomic sequence data from invertebrates (Nässel and Wegener, 2011). However, some species have two homologous genes encoding NPF1 and NPF2, among which have an RYamide C-terminus (Wu et al., 2005a; Roller et al., 2008; Huang et al., 2011; Liu et al., 2013), and NPF1 even produces two splicing variants, NPF1a and NPF1b, which contain RPRFamide at the C-terminus (Garczynski et al., 2005; Roller et al., 2008; Yue et al., 2016). In general, NPF1b is coded by optional intron within the NPF1a coding region. In the present study, one NPF*-*encoding gene was successfully cloned and identified *in D. armandi*, which is in consonance with the results of most insect studies so far. The cDNA sequence of NPF confirms the presence of 133 amino acids, and mature NPF is produced with an amidated RPRFamide C-terminus, which has a typical characteristic of the NPF1 precursor family. All NPF sequences of most insects contain the conserved C-terminal ending RxRFamide (Nässel and Wegener, 2011), which display the form of ancestors in invertebrates from an evolutionary point of view. We also cloned and identified cDNAs encoding NPFR1 and NPFR2 from *D. armandi*, which code a length of 418 and 400 amino acids, respectively. They contained some characteristic residues in the seven transmembrane domains including GN in helix 1, NLX3DX8P in helix 2, SX6IX2DRY in helix 3, WX8P in helix 4, PX7Y in helix 5, FX3WXP in helix 6, and NPX2YX6F in helix 7. This typical pattern shows that the NPFRs belong to the rhodopsin-like GPCR superfamily (Costanzi, 2012). Amino acid sequence identity analysis showed that NPF and NPFRs of *D. armandi* were very similar to that of fellow Coleoptera member *D. ponderosa* and clustered with the Coleoptera group.

We found that both the NPF and NPFRs transcripts were expressed throughout various ages of *D. armandi*, suggesting that the NPF signal system might be involved in regulating some physiological processes in the growth and development of beetles. The distribution and expression level of *D. melanogaster* NPF have indicated that NPF is mainly expressed in the CNS and midgut endocrine cells of larvae and adults (Brown et al., 1999). Furthermore, endocrine cells that produce and secrete NPF were found in some other analyzed insect species, such as *Helicoverpa zea*, *Aedes aegypti*, *Rhodnius prolixus*, *Reticulitermes flavipes*, and *Chilo suppressalis* (Stanek et al., 2002; Gonzalez and Orchard, 2008; Nuss et al., 2010; Huang et al., 2011; Xu et al., 2016). Here, the expression level of *D. armandi NPF* in the midgut is higher than that in the brain of adults and larvae. This is consistent with the situation in *Helicoverpa assulta* and *Helicoverpa armigera* (Liu et al., 2013; Yue et al., 2016). On the contrary, in nymphs of *L. migratoria*, the NPF expression level in the midgut is significantly lower than that in the CNS (Tan et al., 2019). Additionally, the expression of NPF is similar in the midgut and CNS in *Anopheles gambiae* (Garczynski et al., 2005). However, the midgut is not the only part of NPF expression in the digestive system; some previous studies showed the existence of NPF expression in the foregut of the *R. flavipes* and *H. assulta* (Nuss et al., 2010; Liu et al., 2013). Furthermore, significantly higher expression levels of *D. armandi NPF* were observed in female midguts and brains compared to male’s, but this contrasts with the situation in *S. gregaria*, which showed that the expression level of male brains was higher than the female brains (Van Wielendaele et al., 2013). Interestingly, immunocytochemistry indicated that the male adults of *D. melanogaster* showed additional male characteristic NPF expressing neurons, which may be involved in the regulation of circadian rhythm and courtship behavior (Lee et al., 2006). The expression levels of *D. armandi* NPFRs in the head and midgut were in line with obtained data in other insect species (Garczynski et al., 2002; Nässel and Wegener, 2011; Deng et al., 2014; Zhang et al., 2020). For examples, the NPFR expression level was significantly high in the midgut, brain, and accessory nerve of *Drosophila* larvae through *in situ* hybridization experiments (Garczynski et al., 2002). NPFR was highly expressed in the brain, mid-gut, and fat body of *B. mori* larvae, and it was also detected in such parts as the silk gland, malpighian tubule, ovary, and testis (Yamanaka et al., 2008; Deng et al., 2014). In *Rhynchophorus ferrugineus*, NPF corresponding receptor gene was also highly expressed in gut and CNS of eighth instar larvae (Zhang et al., 2020).

In this study, we found that with the prolongation of starvation time, the expression level of NPF in *D. armandi* increased, but the following refeeding caused a continuous drop to the original level. Starvation and re-feeding experiments provided direct evidence that NPF signaling system has an effect on feeding behavior. The result was similar to those observed in other insect species previously. For example, NPF expression levels of *D. melanogaster* larvae were higher in the wandering period in comparison to the following feeding stage period (Wu et al., 2003). Additionally, NPF increased expression in male *Nicrophorus vespilloides* when food-deprived, and the decreased expression of NPFR was detected during parenting (Cunningham et al., 2016). Similarly, in *Apis mellifera*, NPF expression levels were shown to be higher in foragers than nurses, but did not appear to be regulated by nutritional status in the workers (Ament et al., 2011). Furthermore, NPF levels were also demonstrated to be higher in prior to feeding conditions compared to the post-feeding conditions in the hemolymph of *A. aegypti* (Stanek et al., 2002). The expression pattern of NPFR1 in the starvation and re-feeding experiments was consistent with NPF. It is obvious that the expression level of NPFR2 was not significantly changed in starved or refed beetles. These results show that the state of feeding has an influence on the expression levels of NPF and NPFR1 in *D. armandi*, while NPFR2 is more stable under different states. The need for nutrients will increase when beetles are starved. As a result of this, the feeding behavior and food intake might be stimulated in the starvation state. The higher NPF expression level was observed in starved beetles that is likely related to a promotion of appetite in response to starvation.

NPF is a critical factor to manage the selection of food, which regulates the transition between acceptance and rejection of harmful food (Wu et al., 2005a). With overexpression of the *Drosophila* NPF in the CNS of larvae, the feeding time was prolonged, while RNAi-mediated knockdown of the *NPF* gene caused refusal of food (Wu et al., 2003). Furthermore, this is also consistent with the observation that knockdown of NPF or NPFR of *Drosophila* neurons suppresses food attractiveness (Beshel and Zhong, 2013). In this study, we knocked NPF down in adults and larvae by injecting dsNPF and the results showed that this method could effectively inhibit the expression of NPF, with smaller body weight. It has caused the beetles to eat less, leading to a delay in growth and development, resulting in body weight loss. This indicates that silencing of NPF expression in *D. armandi* suppresses their appetite, likely leading to a change in feeding behavior.

The mortality was significantly higher in dsRNA-treated adults and larvae than in the two controls (water-injected and non-injected). Thus, silencing the target gene *NPF* not only inhibited their expression levels but also resulted in augmentation of mortality in the state of both starvation and feeding. The result was consistent with previous studies on *H. armigera*; the NPF RNAi led to high mortality of this species, in which all animals could not pupate or emerge due to abnormal growth and development (Yue et al., 2017).

A previous study reported in *D. melanogaster* that the NPF signal system is regulated by the insulin through the InR/PI3K/S6K pathway (Wu et al., 2005b). In this study, we observed that the *D. armandi* NPF regulates feeding behavior through the effect on energy metabolism, in which downregulation of NPF leads to a decrease of glycogen and an increase of trehalose. Presumably, with less food or even starvation, NPF not only promotes biosynthesis or energy storage but also inhibits metabolism or energy utilization. Feeding behavior provides more nutrients to reduce the metabolic demands of stored glycogen and free fatty acid. This pattern was consistent with *H. armigera* and *Ostrinia furnacalis*, and in contrast to *O. furnacalis*, downregulated NPF causes a decrease of free fatty acid in *D. armandi* instead of total lipid (Yue et al., 2016, 2017), suggesting that the NPF system is involved in insulin signal to regulate energy metabolism, which is a hypothesis that needs to be further investigated.

It should be noted that the regulation of feeding behavior not only is related to NPF but also involves the expression changes of its receptors in invertebrates. In this study, we found that the effect of NPFR1*-*knockdown experiment was consistent with the NPF-knockdown experiment, while there was no significant change in silencing NPFR2 because of the low silence efficiency of dsNPFR2. These results suggest that NPFR has similar functions in the regulation of feeding behavior. However, it is not clear how NPF/NPFR signaling system regulates to modify feeding behavior at the neural circuit, molecular, and cellular levels in *D. armandi*. Further studies will be needed to clarify a more clear explanation of the mechanism involved in the regulation of feeding behavior.

The generation of insecticide resistance is increasingly becoming the primary problem of pest management, and the regulation of the NPF/NPFR signal system is a potential control method. NPF not only is involved in the regulation of many important insect behaviors but also has the characteristics of wide distribution and high conservation, which might become a potential target of new insecticides. In the future research, it is still necessary to further clarify the upstream and downstream action elements of NPF/NPFR, so as to screen out stable and highly active repressors by pharmacology, and finally achieve specific pest control.

## Data Availability Statement

The datasets presented in this study can be found in online repositories. The names of the repository/repositories and accession number(s) can be found in the article/Supplementary Material.

## Author Contributions

BL, MT, and HC designed the experiments and revised the manuscript. BL, DF, HG, HN, and YS preformed the experiments. BL analyzed data and drafted manuscript. All authors read and approved the manuscript for final submission.

## Conflict of Interest

The authors declare that the research was conducted in the absence of any commercial or financial relationships that could be construed as a potential conflict of interest.

## Footnotes

1 http://www.ncbi.nlm.nih.gov/

2 https://www.ncbi.nlm.nih.gov/orffinder/

3 http://www.cbs.dtu.dk/services/SignalP/

4 http://www.cbs.dtu.dk/services/TargetP/

5 http://www.cbs.dtu.dk/services/TMHMM/
