# Supplementary material for: Cloning and Expression of the Neuropeptide F and Neuropeptide F Receptor Genes and Their Regulation of Food Intake in the Chinese White Pine Beetle Dendroctonus armandi
Source: Front Physiol. 2021 Jun 18;12:662651. doi: 10.3389/fphys.2021.662651 (PMC8249871; doi:10.3389/fphys.2021.662651)
Supplement: Supplementary file 1 [file Data_Sheet_1.docx]

**SUPPLEMENTARY MATERIAL**


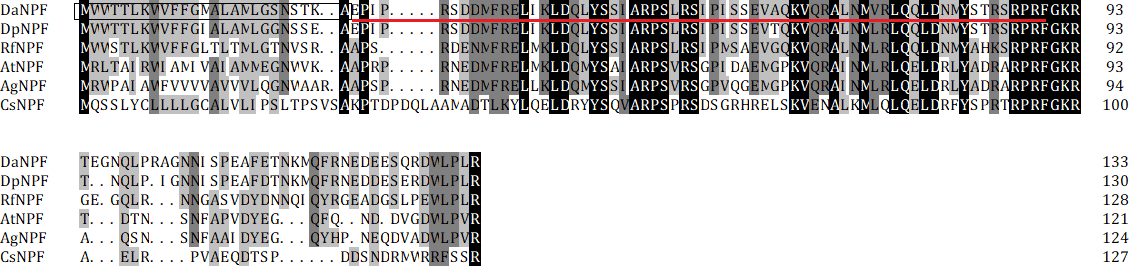


**Figure S1.** Deduced amino acid sequence of *D. armandi* prepro-NPF and comparison of the amino acid sequence of the NPF precursor with those of other species. They include *Dendroctonus ponderosae* (DpNPF), *Rhynchophorus ferrugineus* (RfNPF), *Aethina tumida* (AtNPF), *Anoplophora glabripennis* (AgNPF), and *Cryptotermes secundus* (CsNPF). The putative signal region is indicated by black box, the mature peptide is underlined by a solid red line. Identical amino acid residues in all proteins are shown in black, grey parts indicate similar amino acids.


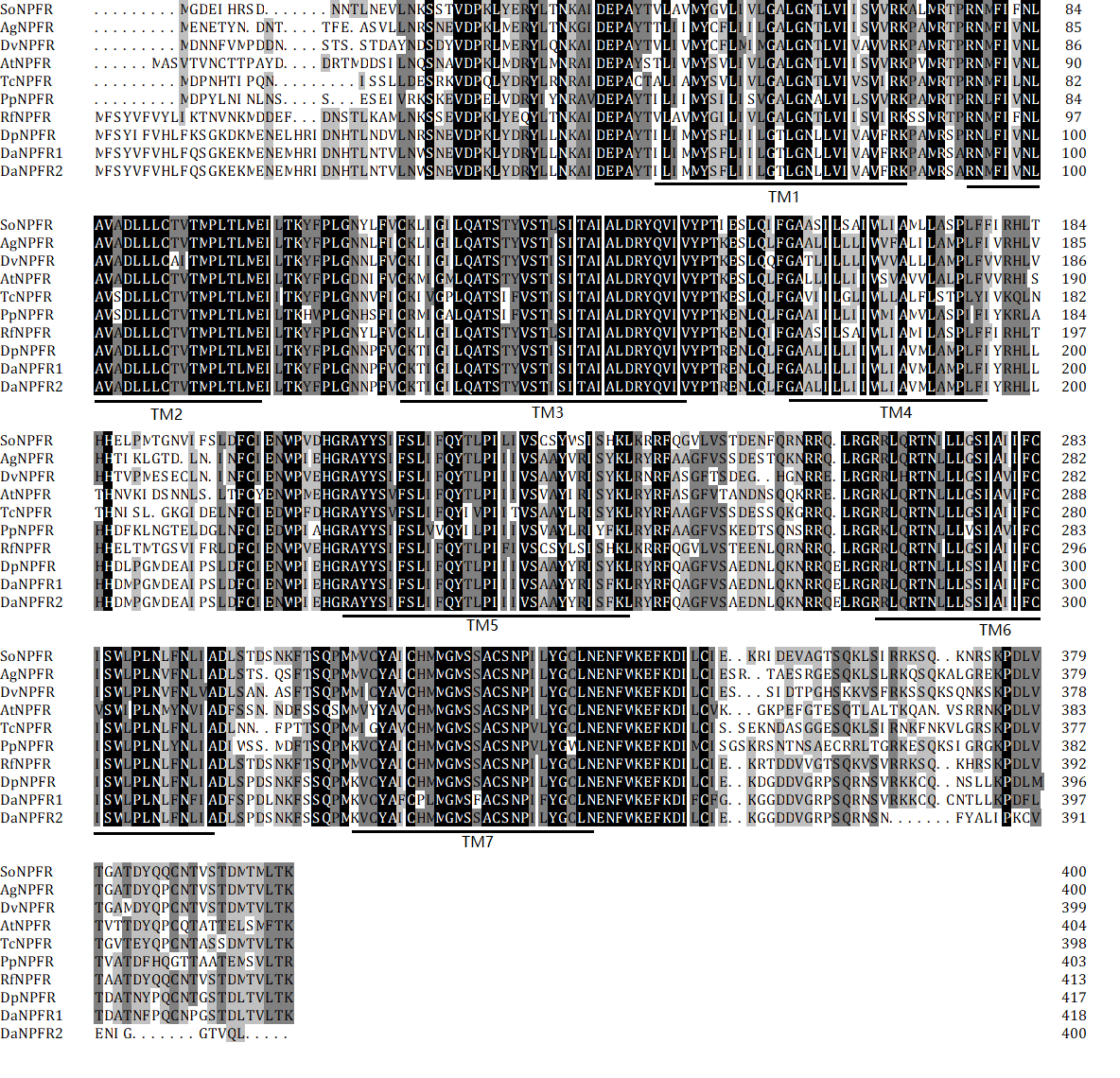


**Figure S2.** Comparison of the amino acid sequence of *D. armandi* NPFRs with those of other species. They include *Dendroctonus ponderosae* (DpNPFR), *Rhynchophorus ferrugineus* (RfNPFR), *Photinus pyralis* (PpNPFR), *Tribolium castaneum* (TcNPFR), *Aethina tumida* (AtNPFR), *Diabrotica virgifera virgifera* (DvNPFR), *Anoplophora glabripennis* (AgNPFR), and *Sitophilus oryzae* (SoNPFR). The predicted seven transmembrane domains are underlined by a solid line. Identical amino acid residues in all proteins are shown in black, grey parts indicate similar amino acids.


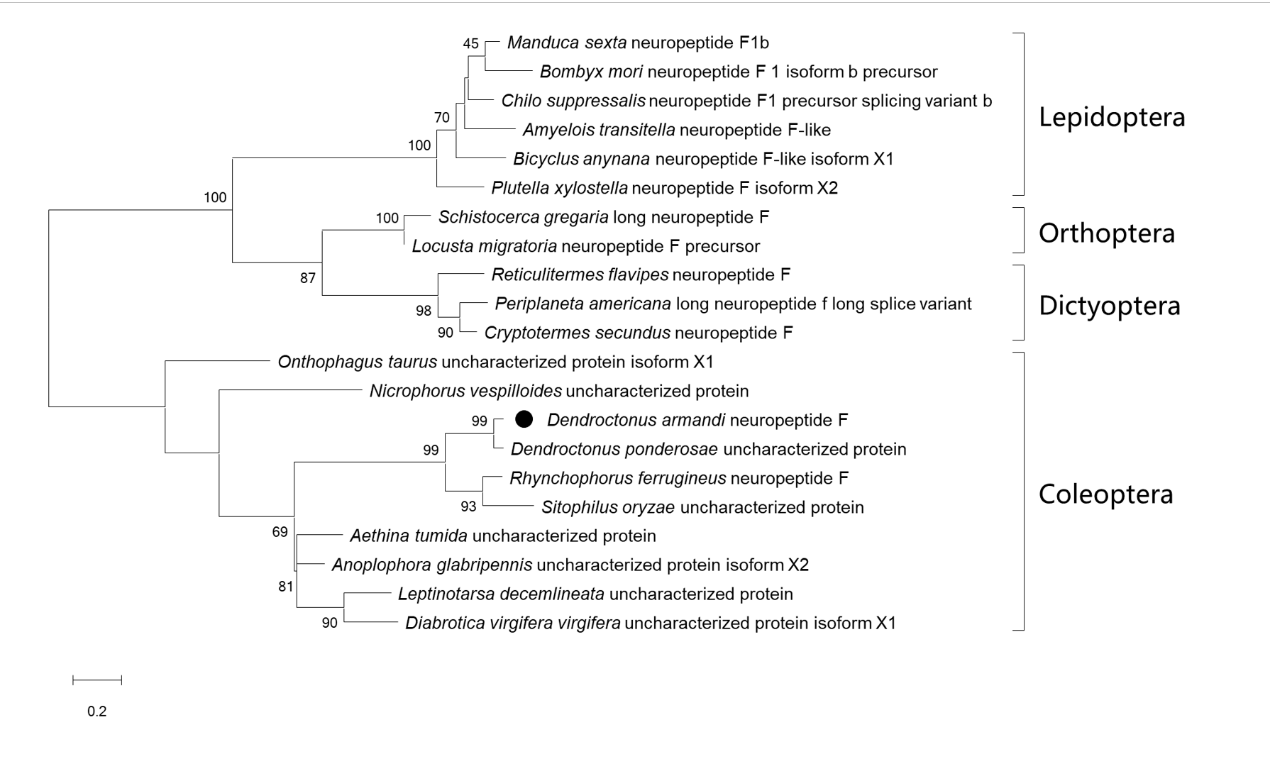


**Figure S3.** Phylogenetic trees based on amino acid sequences of prepro-NPF. The phylogenetic tree constructed by the Maximum Likelihood method using the amino acidic substitution model WAG++G+I+F122 in MEGA5.0. Bootstrap values after 500 pseudo-replicates are shown at nodes. The bootstrap values (in %) are given at each branch point, and below 50% are not shown. The black dot indicates *D. armandi* NPF.


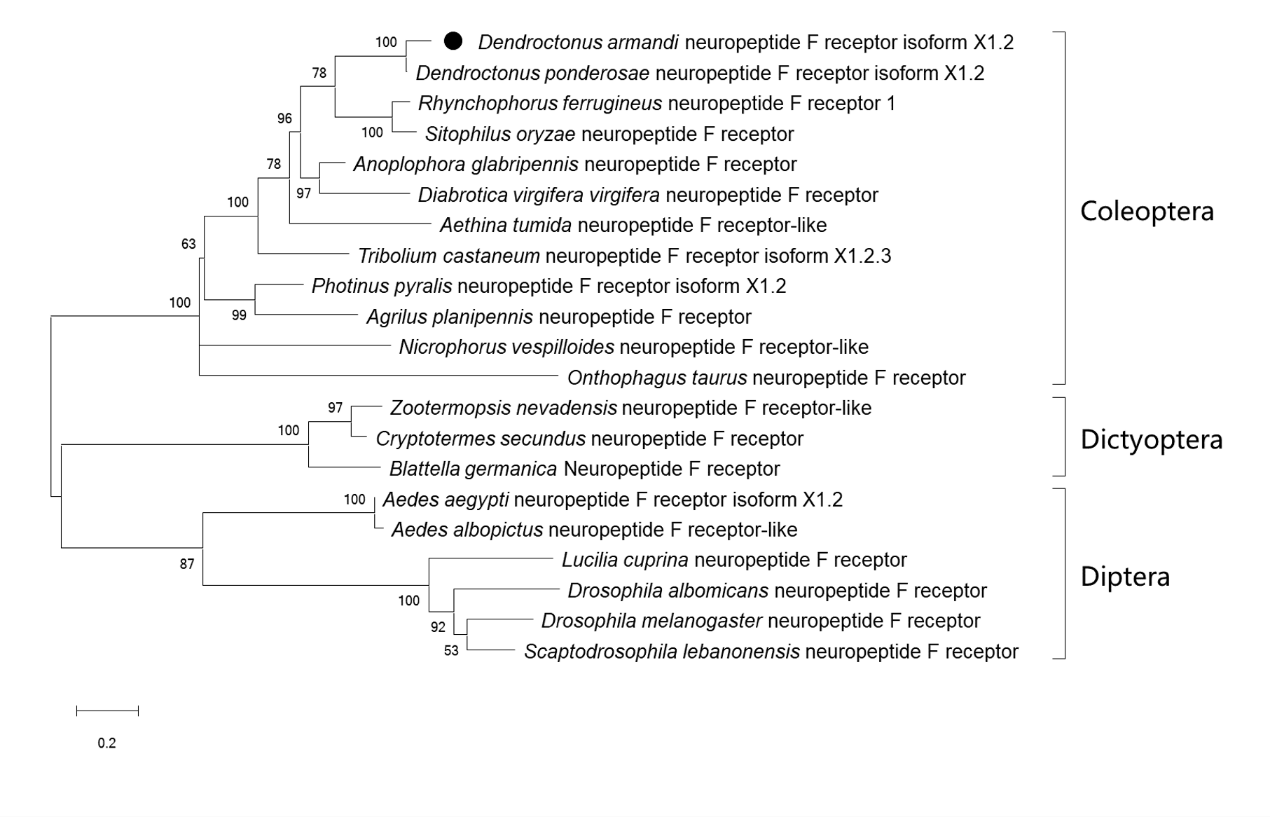


**Figure S4.** Phylogenetic trees based on amino acid sequences of NPFRs from other insect species. The phylogenetic tree constructed by the Maximum Likelihood method using the amino acidic substitution model WAG++G+I+F122 in MEGA 5.0. Bootstrap values after 500 pseudo-replicates are shown at nodes. The bootstrap values (in %) are given at each branch point, and below 50% are not shown. The black dot indicates *D. armandi* NPFRs.

**Table S1** Primer sequences used in the research.

| Gene | Sequence （5′ → 3′） | | purpose |
| --- | --- | --- | --- |
|  | Forward | Reverse |  |
| NPF | GACATGTTCAGGGAGCTA | CTTTCCGAATCTAGGACG | cDNA |
|  | TGTTCAGGGAGCTAATCAAG | GTTGTACTCGTCCATTGCTC | 3’RACE |
|  | GGACGAGCAATGGACGAGTA | GGTAGCCAGTCTCGTTGTGA | 5’ RACE |
|  | GGAAAGCCAGCAGAAGACAT | AGAACTAAACGGGTGATTGT | Full-length |
|  | TCAACCAAGGCAGAACCAAT | TAGCCGCACCATGTTAAGTG | qPCR |
|  | TAATACGACTCACTATAGGG  AAATGGGTCTTCTTCGGGATG | TAATACGACTCACTATAGGG  ACGGTAGCCAGTCTCGTTGT | RNAi |
| NPFR1 | AGAATCGCCGACAGGAGT | AGTTCAAAGGCAGCCAAC | cDNA |
|  | GCATCAGTTGGCTGCCTTTG | GCTGGCACTGCCGTGGTAA | 3’RACE |
|  | TTCAAAGGCAGCCAACTGAT | TTTCCAAAAGTTCTCGTTCA | 5’ RACE |
|  | TTGCCGCTTCGTCGTTTATCA | AAACAACCGAGCTATTCCC | Full-length |
|  | AACGAGAACTTTTGGAAAGAAT | ACACTGTTAAATCCGTACTG  CC | qPCR |
|  | TAATACGACTCACTATAGGG  TGAGGTCGATCCGAAACT | TAATACGACTCACTATAGGG  ACATCACAGCGATTAGCC | RNAi |
| NPFR2 | CGCTTCGTCGTTTATCAG | AAGATTTCCCAATGTGCC | cDNA |
|  | TCGTTCATTTATTCCAATCCGGTAA | TCCTAAATAAAGCCATAGATGAACC | 3’RACE |
|  | TTCAAAGGCAGCCAACTGAT | TTTCCAAAAGTTCTCGTTCA | 5’ RACE |
|  | AGCATTTGAATGTTTTCTTAC | TTTAGTCCATCACATCACAAT | Full-length |
|  | AAATCGGCATCGAACTGAAA | TCCGCATGTAGTAGCCCTTA | qPCR |
|  | TAATACGACTCACTATAGGG  TGCGAACTGTGACCAACTAC | TAATACGACTCACTATAGGG  ACCAAATAACTGCTGCGATA | RNAi |
